# Identification of recurring patterns in fractionated atrial electrograms using new transform coefficients

**DOI:** 10.1186/1475-925X-11-4

**Published:** 2012-01-19

**Authors:** Edward J Ciaccio, Angelo B Biviano, William Whang, Hasan Garan

**Affiliations:** 1 Department of Medicine - Division of Cardiology, Columbia University Medical Center, Columbia University, Harkness Pavilion 804, 180 Fort Washington Avenue, New York, NY, 10032, USA

**Keywords:** atrial fibrillation, catheter ablation, ensemble averaging, pattern recognition, transform

## Abstract

**Background:**

Identification of recurrent patterns in complex fractionated atrial electrograms (CFAE) has been used to differentiate paroxysmal from persistent atrial fibrillation (AF). Detection of the atrial CFAE patterns might therefore be assistive in guiding radiofrequency catheter ablation to drivers of the arrhythmia. In this study a technique for robust detection and classification of recurrent CFAE patterns is described.

**Method:**

CFAE were obtained from the four pulmonary vein ostia, and from the anterior and posterior left atrium, in 10 patients with paroxysmal AF and 10 patients with longstanding persistent AF (216 recordings in total). Sequences 8.4 s in length were analyzed (8,192 sample points, 977 Hz sampling). Among the 216 sequences, two recurrent patterns A and B were substituted for 4 and 5 of the sequences, respectively. To this data, random interference, and random interference + noise were separately added. Basis vectors were constructed using a new transform that is derived from ensemble averaging. Patterns A and B were then detected and classified using a threshold level of Euclidean distance between spectral signatures as constructed with transform coefficients.

**Results:**

In the presence of interference, sensitivity to detect and distinguish two patterns A and B was 96.2%, while specificity to exclude nonpatterns was 98.0%. In the presence of interference + noise, sensitivity was 89.1% while specificity was 97.0%.

**Conclusions:**

Transform coefficients computed from ensemble averages can be used to succinctly quantify synchronized patterns present in AF data. The technique is useful to automatically detect recurrent patterns in CFAE that are embedded in interference without user bias. This quantitation can be implemented in real-time to map the AF substrate prior to and during catheter ablation.

## Background

 Radiofrequency catheter ablation is often used for successful treatment of atrial fibrillation (AF), and is guided in part by the morphology of electrograms recorded from the catheter tip. Of particular interest are complex fractionated atrial electrograms (CFAE), which are composed of multiple deflections with varying baseline, or continuous deflections with low voltage [1]. The CFAE may represent the arrhythmogenic substrate for AF. Ablating CFAE can increase the cycle length of the arrhythmia, suggesting the importance of some of these regions as drivers to maintain AF [2]. Ablation of CFAE may improve outcome of the catheter ablation procedure [3]. Progress in the development of signal processing algorithms to identify CFAE can improve the efficacy of ablation strategies [4,5]. Although AF may originate in the pulmonary veins (PV), in many patients, particularly those with the longstanding persistent type, other areas of the left atrium must be ablated to successfully stop the arrhythmia. However, the precise characteristics of atrial electrograms that would suggest that a particular area should be ablated is currently the subject of debate. Moreover, differing techniques for CFAE quantitation do not necessarily identify the same areas for ablation [6,7]. Furthermore, CFAE identified outside the PVs often represent a large surface area of tissue that when ablated in its entirety, can increase procedure time and the possibility of patient morbidity. Thus the need to recognize CFAE with special quantitative characteristics that when ablated can improve outcome. The presence of morphologic differences and repetitive patterns in CFAE have been observed and quantified as a way to distinguish paroxysmal from longstanding persistent AF patients [8,9]. If such patterns could be distinguished from one recorded sequence to the next, then the substrate could be mapped based on pattern recurrence, which is likely related to degree of arrhythmogenicity [8,9].

 In previous work we described a method of spectral estimation and transformation for analysis of atrial fibrillation data [10-12]. Since this transform is data-driven, the orthogonal basis vectors are unique to the particular data set being analyzed. Thus it is possible to extract the original patterns from which the signals were generated from any additive noise and interference that may be present. As is shown in this study, if a recurrent pattern is present in CFAE, it can be detected by generating and then comparing transform coefficients, and is robust to presence of additive random noise and interference. Pattern recognition techniques are then used to distinguish two recurring patterns from nonpatterns present in the CFAE [13,14].

## Methods

### A Clinical data acquisition

 Electrograms were recorded in a series of twenty patients referred to the Columbia University Medical Center cardiac electrophysiology (EP) laboratory for catheter ablation of AF. These recordings were obtained prospectively as approved by the Internal Review Board at Columbia University Medical Center, but analyzed retrospectively after the catheter ablation procedures were completed using our standard clinical protocols. Ten patients had documented clinical paroxysmal (acute) AF, with a normal sinus rhythm as their baseline rhythm in the electrophysiology laboratory. Atrial fibrillation was induced by burst pacing from the coronary sinus or the lateral right atrial wall, and the arrhythmia persisted for at least 10 minutes for those signals to be included in the retrospective analysis. Ten other patients had persistent (longstanding) AF, and had been in AF without interruption for 1-6 years prior to the catheter mapping and ablation procedure. Bipolar electrograms of at least 10 seconds in duration, recorded from the distal ablation electrode during arrhythmia, were bandpass filtered by the system at acquisition to remove baseline drift and high frequency noise (30-500 Hz), sampled at 977 Hz, and stored. Although the bandpass high corner was slightly greater than the Nyquist frequency, negligible signal energy resides in the region [11].

 Only digitized signals identified as CFAE by two cardiac electrophysiologists were included in the retrospective analysis. The CFAE recordings were obtained from two sites outside the ostia of each of the four PVs. Similar recordings were obtained at two sites on the endocardial surface of the left atrial free wall, one in the mid-posterior wall, and another on the anterior ridge at the base of the left atrial appendage. From each of these recordings, 8.4-second sequences (8,192 sample points) were extracted and analyzed. A total of 240 such sequences were acquired - 120 from paroxysmal and 120 from longstanding AF patients. Subsequently, only 216 of the recordings were confirmed as CFAE, and only these were used for subsequent analysis. All CFAE signals were normalized to mean zero and unity variance prior to further processing [12].

### B Transform coefficients and the spectral signature

 In previous work a mathematical transformation was derived based upon ensemble averaging [12]. For averaging, approximate stationarity of the noise process is assumed. The ensemble average vector ${\underline{e}}_{w}$ of length *w* is then calculated by averaging *n* successive mean zero segments of signal $\underline{x}$ having a length *N*, with each segment being of integer length *w*:

(1a) $${\underline{e}}_{w}=1/n\cdot U_{w}\cdot \underline{x}$$

(1b) n=int(N/w)

The summing matrix is given by:

(2) $$U_{w}=[I_{w}\;I_{w}\;\cdots \;I_{w}]$$

The $I_{w}$ are w×w identity submatrices and are used to form the signal segments of length *w* that are extracted from *x* and summed. Thus ${\underline{e}}_{w}$ is computed by summing segments of the signal having period *w* sample points. The summing matrix $U_{w}$ is padded at right and bottom edge if N/w is not an integer, as described elsewhere [12]. The relationship between frequency *f* and period *w* is given by:

(3) f=sample rate/w

For this study, a range of w=1000−50 sample points (f=0.977 Hz−19.54 Hz) was used for analysis. The power in the ensemble average at period *w* is given by:

(4a) $$P_{w}=1/w\;{\underline{e}}_{w}^{T}\cdot {\underline{e}}_{w}$$

(4b) $$=1/n^{2}w\;{\underline{x}}^{T}\cdot U_{w}^{T}\;U_{w}\cdot \underline{x}$$

The scaling terms 1/w and 1/n account for the *w* summations used to calculate the power, and the *n* summations used to form each ensemble average. The transformation matrix is:

(5a) $$T_{w}=U_{w}^{T}\;U_{w}$$

(5b) $$=\left[\begin{array}{cccc} I_{w} & I_{w} & \cdots & I_{w}\\ I_{w} & I_{w} & \cdots & I_{w}\\ & & \cdots \\ I_{w} & I_{w} & \cdots & I_{w} \end{array}\right]$$

Signal *x* of length *N* can then be decomposed using the linear transformation:

(6) $${\underline{a}}_{w}=1/n\;T_{w}\cdot \underline{x}$$

where ${\underline{a}}_{w}$ are basis vectors, *n* is the number of summations for ensemble averaging, and ${\underline{a}}_{w}$ and $\underline{x}$ are N×1 in dimension. The basis vectors ${\underline{a}}_{w}$ for all *w* characterize the periodic behaviors present in signal vector $\underline{x}$, and they are linearly independent except when small integer relationships exist between periods $w_{i}$ and $w_{j}$ of any particular pair [12]. Columnwise, each identity submatrix in Equation 5b extracts and sums one segment of *w* sample points in $\underline{x}$ (Equation 6), with the sum total being projected onto the canonical basis. Rowwise the identity matrices serve to repeat the ensemble average of length *w* over a total length *N* during construction of ${\underline{a}}_{w}$. Thus the transformation matrix of Equations 5a and 5b decomposes the signals into periodic ensemble averages. These orthogonal basis vectors can be used to project signal $\underline{x}$ into ensemble space:

(7) $${\underline{x}}^{T}\cdot {\underline{a}}_{w}=1/n^{2}w\;{\underline{x}}^{T}\cdot T_{w}\cdot \underline{x}=P_{w}$$

where as in Equation 4b, the inner products are again scaled to account for the total number of summations. Equation 7 states that if each signal segment of length *w* is correlated with the ensemble average at *w* (LHS), the resulting transform coefficient is the ensemble average power at *w* (RHS). The power spectrum can either be plotted versus period *w*, or versus frequency *f*, and to level the noise floor, which depends on the number of summations for averaging, it is scaled by $\sqrt{n}$ when graphed [12]. The transformation process can be analyzed by expanding Equation 7:

(8) $${\underline{x}}^{T}\cdot {\underline{a}}_{w}=1/n^{2}w\;{\underline{x}}^{T}\cdot U_{w}^{T}\;U_{w}\cdot \underline{x}=P_{w}$$

In the middle part of Equation 8, when the mathematical operations are done from right to left, then starting with signal $\underline{x}$, the ensemble average is generated by $U_{w}\;\underline{x}$, orthogonal basis vectors are formed by $U_{w}^{T}\;U_{w}\;\underline{x}$, and transform coefficients by ${\underline{x}}^{T}\;U_{w}^{T}\;U_{w}\;\underline{x}$.

Now consider two signals $\underline{x}$ and $\underline{y}$, such that:

(9) $$\underline{z}=\underline{x}+\underline{y}$$

When the inner product between signal $\underline{z}$ and the basis vectors of $\underline{z}$ is computed:

(10) $$1/n^{2}w\;{\underline{z}}^{T}\cdot U_{w}^{T}\;U_{w}\cdot \underline{z}=P_{wz}$$

it is evident that the transform coefficients are also power spectral coefficients $P_{wz}$, which are nonnegative. By comparison, when the inner product is computed for signal $\underline{x}$ or for signal $\underline{y}$ with the basis vectors of $\underline{z}$, the resulting transform coefficients are not power spectral coefficients since they can be negative as well as positive. Instead they can be signified by coefficients $C_{w}$:

(11a) $$1/n^{2}w\;{\underline{x}}^{T}\cdot U_{w}^{T}\;U_{w}\cdot \underline{z}=C_{wx}$$

(11b) $$1/n^{2}w\;{\underline{y}}^{T}\cdot U_{w}^{T}\;U_{w}\cdot \underline{z}=C_{wy}$$

Thus unlike power spectral coefficients $P_{wz}$, the coefficients $C_{wx}$ and $C_{wy}$ plotted for all *w* are not power spectra. Rather, they represent the power in each basis vector derived from $\underline{z}$ that is correlated to $\underline{x}$ and to $\underline{y}$, respectively. Hence they can be defined as spectral signatures, having similarities to the power spectra of $\underline{x}$ and $\underline{y}$ respectively, but using, for reconstruction, basis vectors that are only partially correlated with each signal. Considering the relationship between $\underline{x}$, $\underline{y}$, and $\underline{z}$, several inequalities should be noted:

(12) $${\underline{x}}^{T}\cdot U_{w}^{T}\;U_{w}\cdot \underline{x}\neq {\underline{x}}^{T}\cdot U_{w}^{T}\;U_{w}\cdot \underline{z}$$

(13) $${\underline{y}}^{T}\cdot U_{w}^{T}\;U_{w}\cdot \underline{y}\neq {\underline{y}}^{T}\cdot U_{w}^{T}\;U_{w}\cdot \underline{z}$$

(14) $$\begin{array}{rcl} & & {\underline{x}}^{T}\cdot U_{w}^{T}\;U_{w}\cdot \underline{x}+{\underline{y}}^{T}\cdot U_{w}^{T}\;U_{w}\cdot \underline{y}\\ & & \;\neq {\underline{z}}^{T}\cdot U_{w}^{T}\;U_{w}\cdot \underline{z} \end{array}$$

As the similarity of $\underline{x}$ and $\underline{y}$ to $\underline{z}$ increases, the inequalities described by Equations 12-14 tend toward becoming equalities. The relationship between the spectral signatures of $\underline{x}$ and $\underline{y}$, and the power spectrum of $\underline{z}$, is given by:

(15) $$\begin{array}{rcl} & & {\underline{x}}^{T}\cdot U_{w}^{T}\;U_{w}\cdot \underline{z}+{\underline{y}}^{T}\cdot U_{w}^{T}\;U_{w}\cdot \underline{z}\\ & & \;={\underline{z}}^{T}\cdot U_{w}^{T}\;U_{w}\cdot \underline{z} \end{array}$$

Equation 15 states that the sum of the spectral signatures of $\underline{x}$ and $\underline{y}$ with respect to $\underline{z}$ equals the power spectrum of $\underline{z}$.

Now suppose that $\underline{z}$ is an average of many signals, some of which contain a particular pattern A or a different pattern B. These two repeating patterns A and B will be reinforced, and the random content will be reduced, by the summation that forms $\underline{z}$. The basis vectors formed from this average will therefore mostly be constructed from a combination of the features from pattern A and those of pattern B. Hence we would expect that if a particular input signal happened to contain pattern A or pattern B, then the resulting transform coefficients $C_{w}$ would be similar to $P_{w}$. In contrast, if the input signal is a random vector unrelated to pattern A or B, the resulting transform coefficients $C_{w}$ for all *w* would approach zero and contain both positive and negative values, due to the lack of correlation of the signal with the basis vectors. These properties can be exploited for detection of two recurring patterns A and B, as described in the next section.

### C Paradigm for recognition of recurrent patterns in atrial fibrillation signals

**Figure 1:**
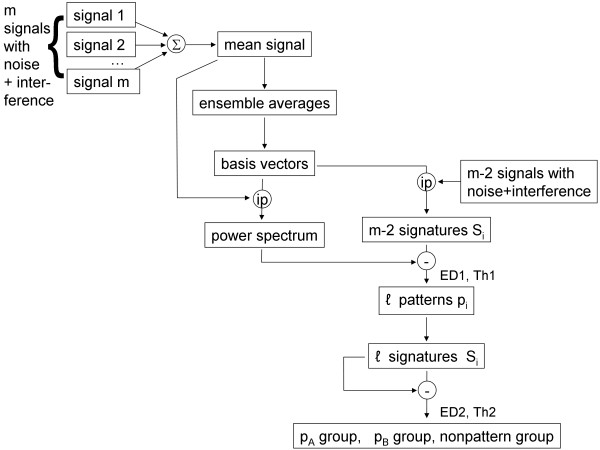
**Flow diagram for pattern recognition in atrial fibrillation signals.**ip=inner product, Σ=summation, −=difference, p=pattern, S=signature, *ℓ*= number of candidate patterns selected by the Euclidean distance threshold value. The number of initial CFAE recordings m=216. After addition of interference, the total number of recordings m−2 is 214. After comparison of the 214 spectral signatures with the power spectrum of the mean signal, based on a first threshold level Th1 for Euclidean distance ED1, *ℓ* candidate patterns are selected. By comparing the spectral signatures of the *ℓ* candidate signals using a second threshold level Th2 for Euclidean distance ED2, selections are made as to whether each candidate contains pattern A, pattern B, or no pattern.

 Patterns A and B described in the last section can be detected as shown in the flow diagram in Figure 1. Consider a set of *m* random signals, a few of which contain pattern A or pattern B embedded in additive random noise and interference, as represented at top left in Figure 1. If the *m* signals are averaged, only recurring patterns will be reinforced in the resulting mean signal (top middle, Figure 1). When the power spectrum *P* is generated from this mean signal (middle portion of Figure 1), it will contain elements of any recurring patterns. If the transform coefficients of each of the *m* individual signals are obtained using the basis vectors constructed from the mean signal, the resulting spectral signatures $S_{i}$ will be similar to *P* only for those signals which contain a recurring pattern. As suggested previously [12], the Euclidean distance (ED) between power spectrum *P* and a particular spectral signature $S_{i}$ will be large if $S_{i}$ lacks a recurring pattern, and small if it contains a recurring pattern. A first threshold level (Th1) of Euclidean distance ED1 can therefore be used to detect the presence of a candidate pattern in $S_{i}$, for all *i* (lower right, Figure 1). Once the candidate patterns in the series are detected, their spectral signatures can be compared one to another using a second Euclidean distance and threshold (ED2 and Th2). The Euclidean distance will be shorter if $S_{i}$ and $S_{j}$ are generated from two signals *i* and *j* containing the same pattern. The Euclidean distance will be longer if $S_{i}$ and $S_{j}$ are generated from signals containing different patterns, or if one or both of the signals contain no pattern. The latter can occur if the first threshold is set to a longer value of Euclidean distance, so that some random signals lacking patterns are initially identified as candidate patterns. Therefore, threshold selection is a tradeoff between excluding actual patterns (shorter Euclidean distance) versus including nonpatterns (longer Euclidean distance). 

The method described above can be automated to detect recurring patterns without manual intervention, except to set threshold level Th1 for pattern detection, and threshold level Th2 to cluster and classify the detected patterns. For the procedure to work, the patterns contained in the signals must be synchronous so that they reinforce upon averaging. This can often be achieved either by simultaneously recording from many atrial sites, or by successive recording using a suitable trigger such as an *F* wave peak when present in the electrocardiogram.

To test the method, signals containing patterns were simulated as follows. Of the 216 CFAE recordings, two were selected at random to be patterns. The first of these, pattern A, was substituted for four other recordings while the second, pattern B, was substituted for three other recordings, selected at random from recording 2 through 215. Thus pattern A was made to occur five times and pattern B four times among the set of m=216 recordings. This set was summed to form the mean signal (top in the block diagram, Figure 1). Interference was then added by combining each signal with the preceding and following signal in the series without replacement, that is:

(16) $$x_{i}=x_{i-1}+x_{i}+x_{i+1},\;i=2,\ldots ,215$$

Of the final series of m−2=214 signals with interferences, 27 thus contained one of two recurring patterns A and B due to the method of combination described in Equation 16. This final set was used for transformation, with patterns A and B detected according to the flow diagram in Figure 1. Since the values of *w* ranged from 50 to 1,000, each spectrum and spectral signature was 951-dimensional. The process was repeated with different patterns selected at random for a total of 10 trials. As an additional test, all of the steps above were repeated with random noise added to each CFAE as well as interference. The random noise vectors with Gaussian distribution were approximately mean zero, and truncated with a standard deviation of ±2 millivolts, twice the normalized CFAE standard deviation.

The sensitivity and specificity of the method was computed separately for CFAE with interference, and for CFAE with interference + random noise, by considering those signals containing a pattern to be positives and those signals lacking a pattern to be negatives. Thus:

(17) $$\begin{array}{rcl} \text{Sensitivity} & = & \mathrm{TP}/(\mathrm{TP}+\mathrm{FN})\\ & = & \text{Correctly identified patterns}/\text{All patterns}\\ \text{Specificity} & = & \mathrm{TN}/(\mathrm{TN}+\mathrm{FP})\\ & = & \text{Correctly identified nonpatterns}/\text{All nonpatterns} \end{array}$$

## Results

**Figure 2:**
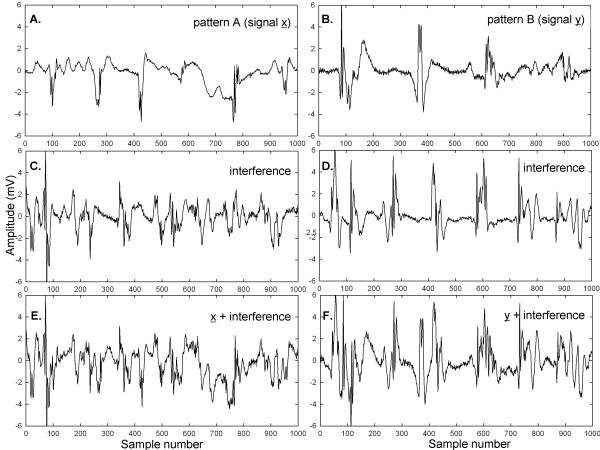
**Examples of atrial electrograms used as patterns A and B to be detected in the set of 216 initial recording sequences (panels (A) and (B)).** When two interferences are added (panels (**C**) and (**D**)) the corresponding original signals are not very discernable (panels (**E**) and (**F**)).

In Figure 2 are presented examples of signals and additive interferences. Identical scales are used in all panels. In panel A is shown a CFAE from the right superior pulmonary vein ostia in a paroxysmal AF patient. In panel B is depicted a CFAE from the anterior left atrial free wall in another paroxysmal AF patient. Both signals have mostly continuous activation, and the large deflections have different shape and timing at each occurrence. Only 1,000 of 8,192 sample points are shown for clarity (approximately 1 second), although 8,192 points were used for the calculations described in the Methods. The signals of panels A and B in Figure 2 were used as patterns A and B, respectively, which were made to occur five and four times, respectively, in the final data set of 214 signals used for analysis. Examples of additive interference are shown in corresponding panels C and D. The interferences are each a combination of two AF signals unrelated to signals patterns A and B. The same patterns after addition of the interferences are shown in the corresponding panels E and F in Figure 2. With the additive interferences, the original signals are almost completely unrecognizable visually. Most of the original signal deflections are masked by interference. 

**Figure 3:**
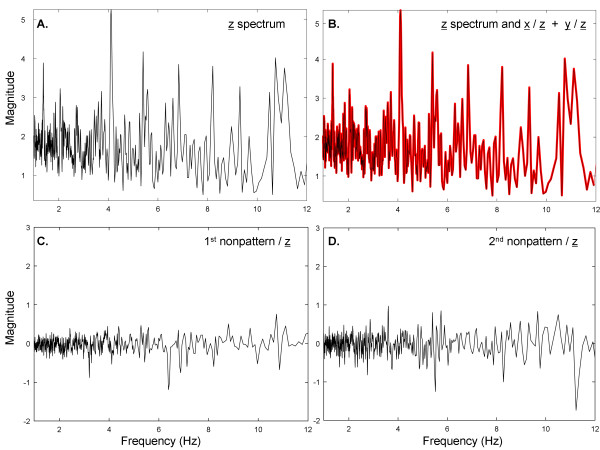
**Example of transform coefficients when two patterns A and B are embedded in interference.** The basis vectors used to compute the transform coefficients were derived from all 216 recordings summed to form a mean signal. (**A**) The spectrum of the mean signal from 216 individual recordings. (**B**) The spectrum of the mean signal *z* (black) and the sum of transform coefficients from pattern A + pattern B (red) are identical and overlapped. (**C**) Transform coefficients for a nonpattern, which is uncorrelated to either pattern A or pattern B. (**D**) Transform coefficients for another nonpattern, which is also uncorrelated to either pattern A or pattern B.

The spectrum of the combined patterns A and B from Figure 2A, B is shown in Figure 3A in the range 1-12 Hz, where pattern A (signal $\underline{x}$) + pattern B (signal $\underline{y}$) form the combined signal $\underline{z}$. Several prominent peaks are present in the spectrum of $\underline{z}$, likely related to individual components of the two signals. The transform coefficients of $\underline{x}$ and $\underline{y}$ with respect to the basis vectors of $\underline{z}$ were separately calculated and then added together and plotted as a red trace in Figure 3B, shown with overlapping $\underline{z}$ spectrum from Figure 3A (black). There is perfect overlap in accord with Equation 15. In contrast, when the spectral signatures of two other signals not related to $\underline{x}$ or $\underline{y}$ are obtained with respect to $\underline{z}$, their magnitude throughout the frequency range is relatively small and the transform coefficients are both positive and negative (panels C and D; same 5-unit range in ordinate scale as in Figure 3A, B). 

**Figure 4:**
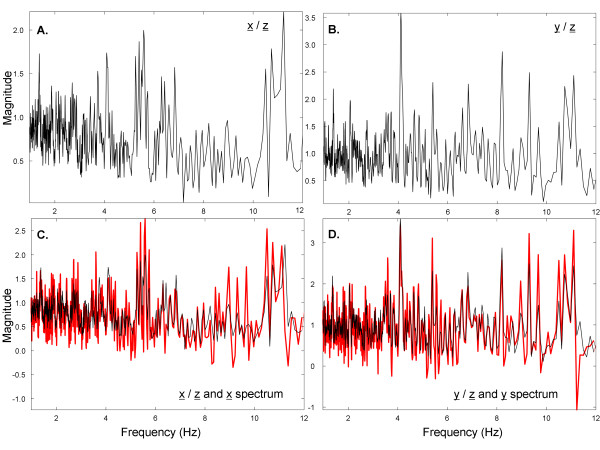
**The spectral signatures of pattern A and B computed from the basis vectors derived from the mean signal.** (**A**) Spectral signature of pattern A. (**B**) Spectral signature of pattern B. (**C**) Comparison of spectral signature of pattern A (black) to spectrum of pattern A (red). (**D**) Comparison of spectral signature of pattern B (black) to spectrum of pattern B (red).

To further elucidate the process, when the spectral signatures of $\underline{x}$ and $\underline{y}$ with respect to $\underline{z}$ are separately plotted (Figure 4A, B, respectively), there are similarities to the $\underline{z}$ spectrum of Figure 3A. Therefore, elements of the $\underline{z}$ spectrum (Figure 3A) are maintained in the spectral signatures of $\underline{x}$ and $\underline{y}$ (Figure 4A, B respectively), suggesting that the Euclidean distances between them will be relatively small. In contrast, the elements of the $\underline{z}$ spectrum are not maintained in the spectral signatures of random interferences such as those shown in Figure 3C, D, suggesting that the Euclidean distances between them will be relatively large. Finally, the spectral signatures of $\underline{x}$ and of $\underline{y}$ with respect to $\underline{z}$, shown again as black traces in Figure 4C, D, are similar, but not the same, as the spectra of $\underline{x}$ and $\underline{y}$, which are denoted as red traces in Figure 4C, D. Based on Figures 3 and 4, the spectral signatures of $\underline{x}$ and $\underline{y}$ with respect to $\underline{z}$ are related to the actual frequency content in signals $\underline{x}$ and $\underline{y}$. However, the $\underline{x}$ and $\underline{y}$ spectra do not resemble each other since they are uncorrelated. 

**Figure 5:**
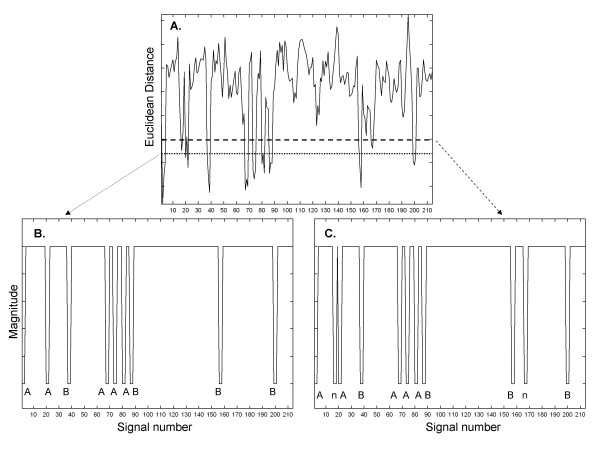
**The Euclidean distance between the power spectrum of the mean from 216 recordings, and the spectral signatures of 214 individual recordings with interference added (panel (A)).** Two thresholds are shown (horizontal lines) which produce different binary functions for recognition (panels (**B**) and (**C**)).

The Euclidean distance between the spectral signatures of each of 214 signals with differing additive interference, versus the spectrum of the mean signal containing two patterns A and B, is shown in Figure 5A. There are a number of downward projections which indicate increased correlation and possible instances of pattern recurrence. If the lower threshold is used, nine possible instances of repetitive patterns are selected (shown in binary form in panel B). When the upper threshold is used, eleven possible instances of repetitive patterns are selected (shown in binary form in panel C). The detected pattern type (A or B) or nonpattern (n) are shown at the bottom of panels B and C. The selection of a threshold higher along the ordinate axis in the Euclidean distance graph of panel A would enable the detection of more candidate patterns. However, whatever threshold is used, to determine and identify the presence of actual recurring patterns necessitates the last step at lower right in the pattern recognition flow diagram of Figure 1, i.e., the spectral signatures of the signals selected by threshold in Figure 5 must be compared. Due to the method of constructing signals plus interference (see the Methods), each downward projection in Figure 5B, C represents a set of three successive signals with pattern, of which the middle was used for statistical calculation. 

The Euclidean distances for all pairings of spectral signatures using the upper threshold in Figure 5A (shown in binary form in Figure 5C) are given in Table 1. The first column and first row in Table 1 note the actual pattern that was selected by the upper threshold in Figure 5, and correspond to the sequence shown in Figure 5C. Since the two patterns A and B occurred only nine times in the sequence, two of the selections in Figure 5, top threshold, were of nonpatterns (n). In the case of the pairing of a spectral signature from a particular signal with itself, the Euclidean distance is zero (main diagonal in Table 1). There is symmetry above and below the main diagonal (half the table is redundant). Smaller values in Table 1 indicate shorter Euclidean distances, i.e., spectral signatures that are more similar. The Euclidean distances tend to be small for spectral signatures of pattern A embedded in one interference versus pattern A embedded in another interference, and similarly for pattern B embedded in one interference versus pattern B embedded in another interference. The Euclidean distances tend to be large for spectral signatures of pattern A versus pattern B embedded in interference, for spectral signatures of pattern A or B embedded in interference versus nonpatterns (interference only), and for spectral signatures of nonpattern versus nonpattern. Thus the patterns and nonpatterns with interference can be distinguished based on a threshold level Euclidean distance. 

**Table 1:** Euclidean distance between candidate patterns

**Pattern**	**A**	**n**	**A**	**B**	**A**	**A**	**A**	**B**	**B**	**n**	**B**
A	0.000	0.142	0.056	0.133	0.092	0.074	0.065	0.128	0.135	0.221	0.135
n	0.142	0.000	0.151	0.149	0.166	0.123	0.131	0.188	0.156	0.143	0.122
A	0.056	0.151	0.000	0.136	0.097	0.068	0.068	0.143	0.145	0.214	0.138
B	0.133	0.149	0.136	0.000	0.167	0.127	0.144	0.101	0.096	0.185	0.063
A	0.092	0.166	0.097	0.167	0.000	0.095	0.101	0.196	0.206	0.271	0.151
A	0.074	0.123	0.068	0.127	0.095	0.000	0.082	0.156	0.124	0.191	0.116
A	0.065	0.131	0.068	0.144	0.101	0.082	0.000	0.156	0.151	0.212	0.152
B	0.128	0.188	0.143	0.101	0.196	0.156	0.156	0.000	0.105	0.241	0.102
B	0.135	0.156	0.145	0.096	0.206	0.124	0.151	0.105	0.000	0.161	0.104
n	0.221	0.143	0.214	0.185	0.271	0.191	0.212	0.241	0.161	0.000	0.168
B	0.135	0.122	0.138	0.063	0.151	0.116	0.152	0.102	0.104	0.168	0.000

A - pattern A, B - pattern B, n - nonpattern. There is symmetry about the main diagonal.

From inspection of Table 1, a threshold level of 0.105 normalized units would be estimative to distinguish patterns and nonpatterns with 100% sensitivity and specificity. Those pairings above 0.105 would indicate that the same pattern is not present on both signals, while pairings less than or equal to 0.105 would indicate the same pattern being present on both signals. Using the threshold 0.105 for clustering and classification in all 10 trials, the results are shown in Table 2, left-hand columns. For 10 trials, the sensitivity to correctly detect and distinguish patterns was 96.2%. The specificity to exclude nonpatterns was 98.0%. For the test of interference + noise, a threshold value for Th2 of 0.132 was found to be efficacious in a test trial, and was then used in all trials. The results are shown in Table 2, right-hand columns, with mean values of 89.1% for sensitivity and 97.0% for specificity. Thus the technique is nearly as efficacious for classification when random noise as well as interference is added to CFAE. 

**Table 2:** Statistics for pattern classification

**Trial number**	**sen: int**	**spe: int**	**sen: int+n**	**sen: int+n**
1	100.0	100.0	91.1	100.0
2	97.8	100.0	97.8	95.0
3	93.3	100.0	82.2	100.0
4	95.6	100.0	88.9	100.0
5	93.3	100.0	88.9	100.0
6	91.1	80.0	88.9	75.0
7	95.6	100.0	88.9	100.0
8	97.8	100.0	91.1	100.0
9	97.8	100.0	84.4	100.0
10	100.0	100.0	88.9	100.0
mean	96.2 ± 3.0	98.0 ± 6.3	89.1 ± 4.1	97.0 ± 7.9

sen - sensitivity, spe - specificity, int - interference, n - noise.

**Figure 6:**
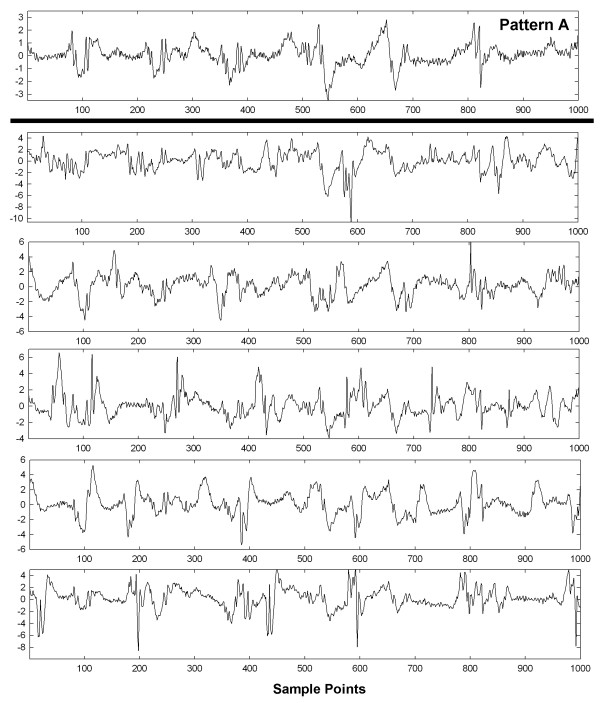
Example of pattern A before interference is added (top graph) and after five different interferences are added (lower panels).

**Figure 7:**
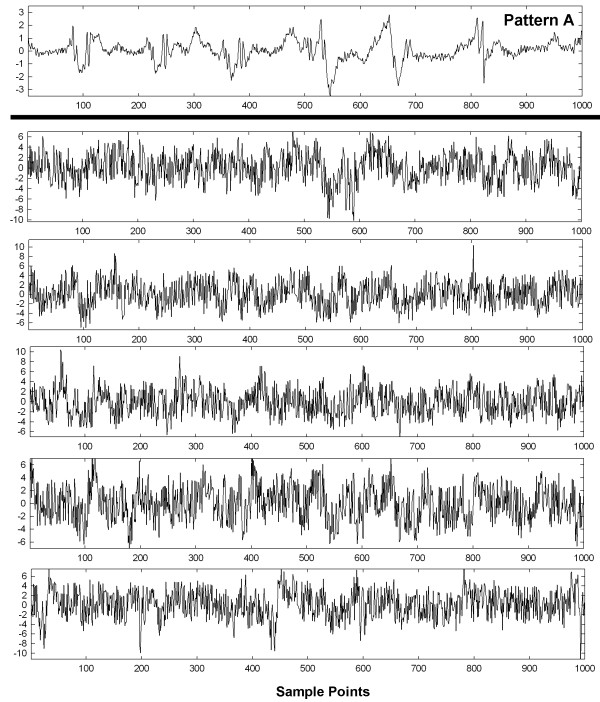
**Example of pattern A before interference+noise is added (top graph) and after five different interferences+noise are added (lower panels).**

**Figure 8:**
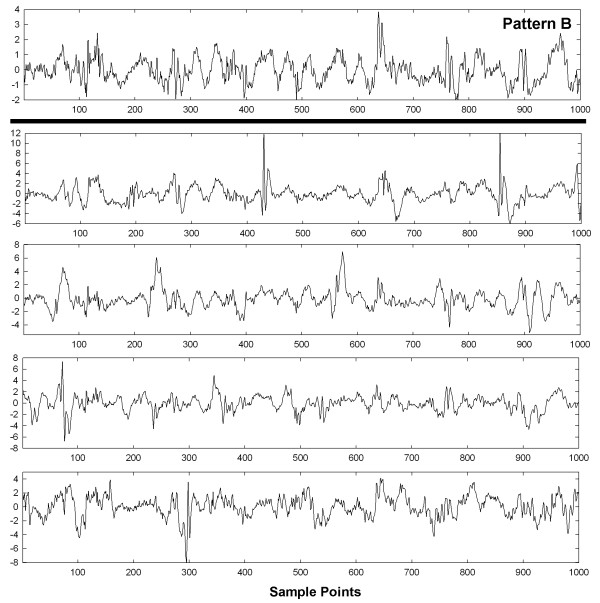
Example of pattern B before interference is added (top graph) and after four different interferences are added (lower panels).

**Figure 9:**
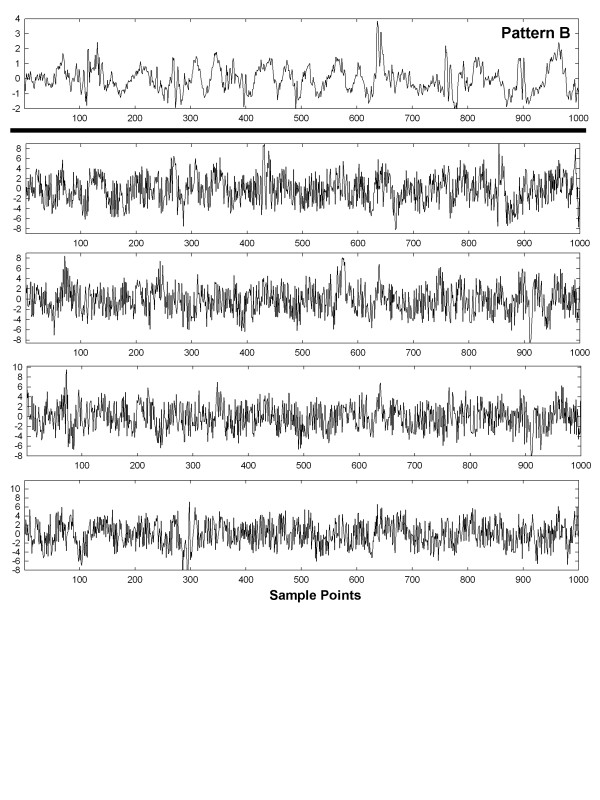
**Example of pattern B before interference+noise is added (top graph) and after four different interferences+noise are added (lower panels).**

**Figure 10:**
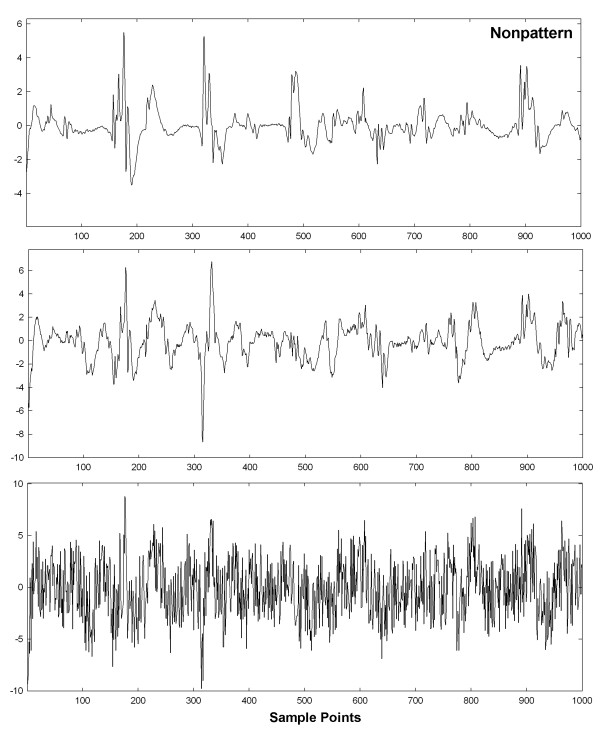
**Example of a nonpattern (top graph), nonpattern with interference (middle graph) and nonpattern with interference+noise (bottom graph).**

To show visually how an entire set of patterns with interference and interference + random noise are affected, graphs of these interactions are shown in Figures 6, 7, 8, 9 and 10 for a selected trial. In Figure 6, pattern A is shown at top for the range 0-1,000 sample points. There are large deflections representing local electrical activation, but these differ in shape and timing from one instance to the next. The five instances of interference added when this pattern occurred among the 214 CFAE sequences are shown in the lower set of graphs. Only a few elements of the original pattern remain recognizable when embedded in interference, such as the large downward deflection between 500 and 600 sample points. In Figure 7, the pattern is repeated in the top graph and in the lower graphs, the pattern is embedded with interference + random noise (same interferences as in corresponding panels of Figure 6). The resulting traces do not appear to be correlated to one another. Similarly, pattern B is shown at top in Figures 8 and 9. In the lower traces in Figures 8 and 9 are shown the four instances of pattern B with additive interference, and with additive interference + random noise, respectively. As for pattern A, there is little evident similarity of these traces to one another or to pattern B itself. Finally in Figure 10 is shown an example of a nonpattern (top), the nonpattern with interference (middle) and the nonpattern with interference + random noise (bottom). The nonpattern is mostly unrecognizable in the lower traces. Overall, the test for distinguishing CFAE patterns with additive interference mostly identified and distinguished traces such as those in Figure 6, lower panels (pattern A), from traces such as those in Figure 8, lower panels (pattern B), from nonpatterns (Figure 10 middle panel). Similarly, the test for distinguishing CFAE patterns with additive interference + noise mostly identified and distinguished traces such as those in Figure 7, lower panels (pattern A), from traces such as those in Figure 9, lower panels (pattern B), and from nonpatterns (Figure 10 bottom panel). 

## Discussion

### Summary

In this study a new transform was used to characterize recurring patterns in CFAE. First, ensemble averages were computed from signal segments of length *w*, repeated for all *w* in the frequency range of interest as given by Equation 3. From each ensemble average an orthogonal basis vector is constructed by repeating the ensemble average of length *w* for the entire signal length *N* (Equation 6). The inner product between basis vector and original signal produces a transform coefficient, which is the signal power at that frequency (Equation 7). The power spectrum is a plot of the entire series of transform coefficients versus frequency. Transform coefficients resulting from the inner product of one signal with the basis vectors of another signal can take on negative as well as positive values, and will have an average level near zero if the signals are uncorrelated (Equations 11a and 11b). The correlation coefficients formed from correlated signal $\underline{x}$ with the basis vectors of $\underline{z}$, as described in the Methods, can be similar to the spectrum of $\underline{x}$ and is termed the spectral signature. Transform coefficients were used to detect two recurring patterns in a sequence of CFAE, embedded in interference and random noise, and to distinguish them from each other and from nonpatterns. The method was implemented and repeated for 10 trials. No manual intervention was used except to set initial threshold levels of Euclidean distance for identification of correlated content, i.e., for pattern extraction, and to distinguish the extracted patterns (Figure 1).

### Prior work in pattern recognition

 Our study made use of correlation in the frequency domain to discern repetitive patterns from nonpatterns in CFAE. Electrogram pattern recognition using correlation waveform analysis has also been found useful to discern electrograms arising from arrhythmia in another study [15]. In this prior work, the electrogram shape at any particular recording site was shown to remain stable during electrophysiologic study, even during such interventions as overdrive pacing and infusion of pharmacologic agents, which included epinephrine and isoproterenol [16]. Thus we expect that our method for detecting and discerning patterns in CFAE will likely be robust to the typical interventions that are done during the course of a clinical electrophysiologic study. We also found the transformation to the frequency domain to be useful to characterize each signal and its relationship to the mean signal. Similarly, in a prior study the wavelet transform was found useful to discern atrial electrogram patterns by categorizing them into one of four classes of fractionation based on frequency and phasic relationships [4,5]. Although the patterns used in our study were synchronized artificially for recognition, multielectrode recordings will likely be useful to simultaneously acquire data from many recording sites in which any patterns present will be synchronized. This would simplify the mapping procedure using the paradigm of Figure 1. Elsewhere, it has been shown that multiple simultaneously obtained recordings are indeed useful for rapid and accurate classification of CFAE patterns [16,17]. Although we have not yet proposed a paradigm to relate the detected patterns to the electrophysiologic substrate, it is planned to develop such a technique for a future prospective study. Adopting a standardized description of CFAE morphology and use of reproducible methodology would enable ease of comparison between clinical trials [17].

 Regarding the possible relationship of observed pattern to electrophysiologic properties of the substrate, the complexity of CFAE, as determined by pattern type, is believed to be related to the degree of organization of electrical activity [18,19]. To reduce complexity, adaptive template matching can be used to normalize the signals with respect to atrial cycle length (*x*-scale), as well as amplitude differences (*y*-scale) [20]. This would be useful to compare patterns in persistent versus paroxysmal AF independent of the dominant frequency, which tends to be higher in the persistent type, and to compare regions of atrial fractionation having low electrogram amplitude to areas with higher electrogram amplitude [11].

### Potential advantages of the new method

The spectral signature is a graph of the correlated content between two signals in frequency space, which can be exploited for pattern recognition (see the Methods). If a series of signals is averaged and basis vectors of the mean are used to obtain the spectral signature of each individual signal, then there will be correlation between the spectrum of the mean, and the spectral signature of the individual signal, when the individual signal contains a synchronous pattern that recurs within the series. By measuring the Euclidean distance between all individual signals having spectral signatures similar to the power spectrum of the mean signal, patterns contained in the sequence can be identified, distinguished from one another, and distinguished from nonpatterns when the nonpatterns are mostly uncorrelated with respect to the mean signal. Thus the new technique is potentially useful to automatically identify and distinguish repetitive patterns present in a series of signals, once threshold levels for the Euclidean distance estimate to detect candidate patterns, and to discern patterns, are established. In the latter step, if multiple patterns are present they can also be discerned using a single threshold level, since the Euclidean distance will be short only with respect to members of the same class.

We found that best threshold levels are sensitive to the degree of additive random noise and interference, and differed when only interference was added versus interference + random noise (0.105 versus 0.132, respectively). Once the threshold levels were established based on a test trial, they were much less sensitive to particular patterns and patients i.e., the results for each trial were similar (Table 2).

### Clinical correlates

 When considering the potential for recurrent patterns to appear in CFAE signals, fractionated electrogram deflection morphology is probably mechanism-dependent. Possible sources of fractionation include areas of slow electrical activation, wavefront collision, anchor points at regions driven by a reentrant circuit, and presence of multiple activation wavelets as triggered, for example, from ganglionated plexi [1]. These mechanisms are potentially distinguished both by spatiotemporal occurrence, and as described in this study by the frequency characteristics of the actual patterns. When using an automated technique for pattern detection, user bias is eliminated as a variable for defining ablation targets [1,13,14]. A goal of CFAE pattern recognition software is to visualize the spatial distribution of CFAE for catheter ablation [21]. Our technique can be used for spatial mapping of CFAE by type of pattern present at each recording site. CFAE are observed at both PVs and the left atrial free wall, although their occurrence at the free wall is more common in persistent AF [21]. Acute and longstanding persistent AF were shown by our group to have CFAE that differ in morphologic characteristics and degree of repetitiveness [8,9]. Therefore, patterns in CFAE and their frequency of occurrence are likely to differ by AF type, and to present differently during mapping procedures. Although in previous work, recurring features in the form of individual deflections were measured on the order of tens of milliseconds in duration, in the current study, patterns in the data were measured over the entire sequence length used, 8,192 sample points (8.4 s). Thus the new technique assumes statistical stationarity over this time interval. Indeed, it has been shown in a previous study that 8-second lengths are ideal for characterizing the dominant frequency of CFAE [22]. Use of a noncontact catheter during clinical electrophysiologic study would enable simultaneous recording of CFAE from multiple sites. From the simultaneously recorded CFAE, far-field electrical activation patterns originating from distant drivers can be detected and localized [23]. Analysis of atrial electrogram patterns using a noncontact catheter is planned for future work. Recurring patterns in data obtained from sequential recording sites using a standard catheter may also be detectable when synchronized by a trigger such as the peak of the *F* wave in the electrocardiogram, also planned as a future study.

### Conclusions

 A method was developed to recognize recurring patterns in AF data using a data-driven transform. It was shown to be efficacious for detection of recurring patterns with additive interferences and noise, simulated from actual data obtained from paroxysmal and persistent AF patients. Although the additive interference and noise rendered the original patterns almost unrecognizable by visual observation (Figures 2,6,7,8 and 9), the spectral signatures of each signal with additive interference and random noise, as related to the correlated content in the mean signal, were useful for detecting patterns, for distinguishing between two different patterns that were present, and for discerning patterns from nonpatterns using two threshold Euclidean distances. This paradigm may also be useful to develop a mechanistic understanding of paroxysmal and persistent AF, because presence of recurrent patterns can be compared and contrasted between the two AF types. It may also be useful to apply to other types of biomedical data such as ventricular tachyarrhythmias, and to videocapsule images of the small intestine, where spectral estimation from signal averaging has been described previously [24,25].

### Limitations

 Although the method described in this study was accurate for detecting simulated recurring patterns embedded in interference, real patterns may differ from one instance to the next, for example as caused by spatial and temporal jitter, which can reduce sensitivity and specificity [11]. Furthermore, the technique was shown to be useful for synchronous pattern data only. If the patterns are highly out of phase, they will not be reinforced when the mean signal is formed (Figure 1), and thus will not appear as correlated content. Therefore, synchronized pattern data is necessary for accuracy, as would more likely be effected by acquiring individual signal data from many recording sites simultaneously, or by synchronizing successively recorded data, for example by triggering the onset of the atrial recording to the presence of an *F* wave in the electrocardiogram.

## Competing interests

The authors declare that they have no competing interests.

## Author contributions

EJC developed the mathematical transform, conducted the data analysis, and wrote the manuscript. ABB, WW, and HG made helpful suggestions, provided the clinical data, and determined which recordings were complex fractionated atrial electrograms. All authors have read and approved the final manuscript.
